# Effects of composition, thermal, and theological properties of rice raw material on rice noodle quality

**DOI:** 10.3389/fnut.2022.1003657

**Published:** 2022-09-02

**Authors:** Ping Wei, Fang Fang, Guoming Liu, Yayuan Zhang, Linyan Wei, Kui Zhou, Xiangrong You, Mingjuan Li, Ying Wang, Jian Sun, Sili Deng

**Affiliations:** ^1^Agro-Food Science and Technology Research Institute, Guangxi Academy of Agricultural Sciences, Nanning, China; ^2^Guangxi Key Laboratory of Fruits and Vegetables Storage-processing Technology, Nanning, China; ^3^Whistler Center for Carbohydrate Research and Department of Food Science, Purdue University, West Lafayette, IN, United States; ^4^Guangxi Institute of Botany, Guangxi Zhuang Autonomous Region and Chinese Academy of Sciences, Guilin, China

**Keywords:** rice, rice noodle, processing, cooking performance, sensory properties

## Abstract

The study aims to evaluate the relationships between characteristics of regional rice raw material and resulting quality of rice noodles. Four of most commonly used rice cultivars in Guangxi for noodles production were investigated. The results showed that compositions of rice flour primarily affected gelatinization and retrogradation, which then influenced the textural and sensory properties of rice noodles. Amylose content had strong positive correlation with peak viscosity (PV) and trough viscosity (TV) of rice flour (*P* < 0.01). PV and TV had strong negative correlations with adhesive strength (*P* < 0.01) and positive correlations with chewiness (*P* < 0.05), hardness, peak load and deformation at peak of rice noodles (*P* < 0.01). Protein content had positive correlation with the Setback of rice flour (*P* < 0.05), which is known to have influences on retrogradation. In addition, solubility had positive correlations with cooking loss (*P* < 0.01) and broken rate (*P* < 0.05) of rice noodles and strong negative correlation with its springiness (*P* < 0.01). Swelling power had negative correlation with broken rate (*P* < 0.05). As sensory score of rice noodles was negatively correlated with broken rate (*P* < 0.05) and cooking loss (*P* < 0.01) and positively correlated with springiness (*P* < 0.01), solubility and swelling power of rice flours were presumed to be useful for predicting consumer acceptability of rice noodles.

## Highlights

- Amylose content of rice is strongly correlated with the texture profile of rice noodle.
- Adhesive strength had a negative correlation with texture profile of rice noodles.
- Peak, trough, and final viscosities had positive correlations with texture of rice noodles.
- Rice with 24% amylose content could be a critical value used in different rice noodles processing.
- Sensory score correlated negatively with cooking qualities and positively with springiness.

## Introduction

Rice noodles are very popular in southern China and some southeast Asian countries, such as Thailand and Sri Lanka. Rice noodles are commonly processed from indica rice, and classified into fresh, dried, and frozen products in various thicknesses and shapes (1, 2). Guangxi province is the top producer of rice noodles in China and has a large number of different types of rice noodles. Consumers have different requirements regarding taste and mouthfeel properties for different types of rice noodles. For example, snail noodles have relatively high hardness and springiness, while rolled noodles and Guilin rice noodles have soft texture.

Rice noodle quality is closely related to the physical and chemical properties of rice flour. Starch is the major component of rice. Just like other crop starch (3–5), the viscoelastic property of rice noodles depends primarily on the starch structure and properties (2, 6, 7). Zhou et al. (8) reported a highly significant correlation between amylose content and the sensory score of rice noodles, so amylose content was usually selected as the sensitive indexes to predict the quality of rice noodles. The amylose content suitable for processing the pressed fresh rice noodles were found to be in the range of 22.2–26.9 %. Xuan et al. (9) suggested that it is essential to use rice with an amylose content in the range of 20–25% for rice noodle production. Rice with amylose content of less 20% or more than 25% is not suitable for rice noodle production. It can be seen from the above that the recommended range of amylose content for rice noodle production by different researchers was different. The appropriate rice starch content should be selected according to the actual demand of rice noodle products. Rice protein (endosperm protein) is also an important component of rice. The protein content in rice varied greatly in different varieties growing in different environments. Martin et al. (10) suggested that the gelatinization characteristics of rice flour were influenced by the network structure formed by the protein binding with water and the formation of disulfide bond. Baxter et al. (11) concluded that rice protein indirectly affected the rice processing adaptability mainly by changing gelatinizing properties of starch, including its heat resistance, extrusion performance and retrogradation. Protein in rice could inhibit the water absorption and expansion of starch particles, resulting in higher gelatinization peak temperature of rice flour than that of starch alone. In addition, protein could strengthen the network structure of rice flour gel. Therefore, it is often considered that protein content can be used as an auxiliary index to choose the rice flour raw material for noodle production. The lipid content in rice starch is very low, but it is closely related to the gel properties of rice. Ibáñez et al. (12) reported that lipids had a greater effect on gelatinization and rheological properties compared to protein. Usually, the presence of lipids reduces the gelatinization heat enthalpy of starch and promotes the formation of gel system. The complex formed by lipids and starch prevents the amylose crystallization, reduces the dissolution of starch, maintains the stability of gel structure, and thereby inhibits the generation of aging and extends the shelf life of products. As lipid content in all kinds of rice varieties were usually very low, most previous reporter considered that it is no longer considered as an important quality index of raw rice used for rice noodles (line) production.

Different rice noodles are commonly produced from different sources of raw rice materials (mainly refer to the early polished indica rice), which is largely depends on the experiences of rice producer using regional rice varieties as raw materials. For example, dry rice noodles are usually made with Guichao varieties; some other rice varieties are combined with broken rice for snail rice noodles production; and rolled noodles are usually made from Zhengui varieties in Gxuangxi. It is very important to clarify the relationship between physical properties of rice varieties and the quality of regional rice noodles in China, which thereby could better guide the production of high-quality regional rice noodles. In the present study, four rice cultivars commonly use in rice noodle production in Guangxi were chosen to prepare rice flours. Their physicochemical composition, physicochemical characteristics, thermal properties, pasting properties, and rheological properties were investigated. Furthermore, the correlation between the physicochemical properties of rice starch and qualities (cooking qualities, texture properties and sensory quality) of rice noodles were analyzed. This study provides theoretical guidance regarding the selection of suitable rice raw materials for different rice noodle production, optimizing rice noodle processing technology, and improving rice noodle quality.

## Materials and methods

### Chemicals and reagents

Four early polished indica rice cultivars, including Zhengui (ZG), Shuanggui (SG), Guichao (GC), and Suimi (SM, belongs to GC variety) were selected and purchased from a local market in Nanning, China. Rice was ground by a high-speed grinder (WND-200, Zhejiang Lanxi Weinengda Electric Appliance Co., Ltd., Lanxi, China), passed through a 100-mesh sieve (CT410, FOSS Scino Co., Ltd., Suzhou, China), and then stored at 25°C in desiccator until further analysis. Amylose contents in rice standards were 0.40, 10.60, 16.20, and 26.50% w/w, respectively, which was provided by China National Rice Research Institute. D-Glucose, α-amylase, and glucosidase were purchased from Sigma-Aldrich Ireland Ltd. (Dublin, Ireland). GOPOD was obtained from Megazyme (Bray, Ireland). Potassium hydroxide (cat. no.1310-58-3), sodium sulfite (cat. no.7757-83-7), sodium hydroxide (cat. no.1310-73-2) were from Sinopharm Chemical Reagent Co., Ltd. ethanoic acid (cat. no. 64-19-7), ethyl alcohol (cat. no. 64-17-5), acetic acid sodium salt (cat. no. 127-09-3), iodine (cat. no.7553-56-2), potassium iodide (cat. no.7681-11-0), ethanoic acid (cat. no.64-19-7) were from Shanghai anpu Experimental Technology Co., Ltd. All chemical reagents were of analytical grade.

### Properties of rice flour

#### Compositions of rice flour

The moisture content, crude protein, and crude lipid contents in different rice flours were measured according to the method of Ministry of Health of the People's Republic of China (GB5009.3-2016, GB/T 5009.5-2016 and GB/T 5009.6-2016) respectively (13–15). The content of total starch in rice was determined using the method (AOAC, 996.11) provided by the Association of Official Agricultural Chemistry (16). The amylose contents were determined by measuring the absorbance at 700 nm via UV-Vis spectrophotometer (UV-2800, Unico Instrument Co., Ltd., Shanghai, China) following the method of Ministry of Health of the People's Republic of China (17). The adhesive strength of different rice flours was evaluated according to the method of Ministry of Health of the People's Republic of China (18). The chemical compositions of rice were determined in triplicate for each rice sample, and all results were reported on a dry weight basis.

#### Solubility and swelling power of rice flour

The solubility (S) and swelling power (SP) were determined following the method as described by Yi et al. (19) with minor modifications. Rice flour (1 g) in 100 mL deionized water was heated at 90 °C for 1 h with stirring. The sample was cooled to room temperature and centrifuged at 4,000 r/min for 15 min. The supernatant was dried in an oven at 105 °C until a constant weight was obtained. The S and SP were calculated using the following formulas:


$$\begin{array}{lll} \text{S}(\%) & = & \text{dry supernatant weight}/\text{dry sample weight}\times 100\\ \text{ SP} & = & \text{wet sediment weight}/[\text{dry sample weight}\times (1-\text{S})] \end{array}$$

#### Thermal properties of rice flour

Thermal properties of the rice flour were analyzed using a differential scanning calorimeter (DSC, TA Instruments, Q2000, USA). Rice flour and distilled water suspension (1:3) were sealed in aluminum pans and equilibrated at room temperature for 24 h before analysis. An empty aluminum pan was used as a reference. The gelatinization temperature and enthalpy were determined following the procedure of Wu et al. (20) with some modifications. The samples were heated at 10 °C/min over a temperature range of 30–105 °C. After that, the sealed pans were stored at 4°C for 7 d, followed by characterization of retrogradation with the same heating procedure. The onset temperature (To), peak temperature (Tp), conclusion temperature (Tc), enthalpies in gelatinization (ΔH_1_) and retrogradation (ΔH_2_) were recorded. The retrogradation degree (ΔH) was calculated as follows:


$$\begin{array}{l} \text{Δ}H/\%=\frac{\text{Δ}H_{2}}{\text{Δ}H_{1}}\times 100 \end{array}$$

#### Pasting properties of rice flour

The pasting properties of the rice flours were determined using a Rapid Visco Analyzer (RVA 4800, Perten Instruments Australia Pty Ltd., Sydney, Australia). The RVA parameters were previously described by Geng et al. (21). Rice flour (3.0 g, 14 g/100 g moisture basis) was weighted into an aluminum canister, and 25 g distilled water was added to attain a total sample weight of 28.0 g. The suspension was equilibrated at 50°C for 1 min, heated to 95°C at a rate of 12°C /min and maintained at 95°C for 2.5 min. It was then cooled to 50°C at the same rate and maintained at 50 °C for 2 min. The rotating speed of the paddle was kept at 160 rpm throughout the measurement. The parameters including Peak Viscosity (PV), Trough Viscosity (TV), Breakdown (BD), Final Viscosity (FV), and Setback (SB) values were obtained.

#### Rheological properties of rice flour

Rheologic properties of all samples were analyzed following the method as described by Meng (22) and carried out using a Discovery-1 rheometer (DHR-1, TA Instruments Ltd., New Castle, DE, USA). Rice flour suspensions (20%) were placed in the center of a Peltier plate attached to the rheometer. An aluminum parallel plate geometry (40 mm diameter) was used. The gap was set at 1,000 μm. Oscillation temperature ramp tests were performed at a strain of 2%, with a temperature ramp from 25 to 95°C to gelatinize the sample, followed by cooling down from 95 to 25°C for gel formation. The heating and cooling rates were both 5°C/min. Subsequently, frequency sweep tests were performed at 25°C in the range of 0.1–20 Hz at a strain of 2%. The 2% strain was in the linear viscoelastic region, according to the strain sweep results. Rheological parameters such as storage modulus (G′) and loss modulus (G″) were obtained directly from rheometer data analysis software (Trios.Version 4.3.0.38388).

### Rice noodles

#### Preparation of different rice noodles

Rice noodles were prepared using a One-step Modeling Rice Noodle Machine (MFD25C, Hunan Fenshifu Mechannical Technology Co., LTD, China). Before extrusion, rice was soaked in water at room temperature for 8 h. The soaked rice was poured into the extrusion pipe (preset temperature at 90°C) and extruded through a circular die with 0.20 cm round openings, and then and cut to the same length as they exited the extruder die. After extrusion, the rice noodles were placed in an incubator (YH0515T, Hunan Fenshifu Mechannical Technology Co., LTD, China) for 4 h at 75% humidity and 28°C to allow starch retrogradation to some extent. Finally, the rice noodles were taken out and dried at room temperature for 12 h to reduce the moisture content to below 14%. The dried noodles were kept in sealed plastic bags before analysis.

#### Cooking qualities of different rice noodles

The cooking time and broken rate of different rice noodles were determined by AACC standard methods (23). Five g of rice noodles with length of 15 cm for each strand were placed into 150 mL of boiling water and cooked. Cooking time was determined as the time required for the disappearance of white core as judged by gently squeezing the noodle between two glass slides. Broken rate was the ratio of the number of broken noodles to that of dried noodles. Noodles were drained, and the cooked water was collected in a beaker. The solid material content in the cooking water was determined by evaporating in a hot air oven at 105 °C overnight until a constant weight was reached. The cooking loss of noodles were determined according to Raungrusmee et al. (24) by the following equations:


$$\begin{array}{l} \text{Cooking loss/}\%=\frac{\text{remaining solid content after drying}}{\text{weight of fresh noodle}}\times 100 \end{array}$$

#### Sensory evaluation of rice noodles

The sensory properties of rice noodles were evaluated on a percentage point system (< 60 means poor, 61–80 means intermediate level, >80 means excellent) according to Wang et al. (25) with some modifications. The sensory panel was composed of 10 trained members who were 25–35 years old (five men and five women). All the cooked rice noodles were coded with random four-digit numbers. Meanwhile, water was provided for the panelist to gargle before testing different rice starch noodles. The samples were evaluated using a 100 point scale and the sensory characteristics include color (0–15 points), odor (0–10 points), tissue shape (0–15 points), firmness (0–20 points), smoothness (0–20 points) and elasticity (0–20 points), The value of each sensory characteristic was averaged and the total points were expressed as the sum of all sensory characteristics scores.

#### Textural properties of rice noodles

The textural profile of rice noodles was evaluated by texture profile analysis (TPA) and tensile properties using a texture analyzer (CT3, Brookfield, USA) according to a reported method (26). The rice noodles were cooked in boiling deionized water for the best cook time, followed by cooling to room temperature with deionized water, and drained off the water before measurement. For TPA, noodle samples were cut to 10 cm segments. There segments were placed in parallel with no space on the groove of the plate. The measurement parameters of TPA were: TA5 cylinder probe (diameter 12.5 mm and length 35 mm) at the test speed of 2.0 mm/s, 50% compression ratio, 5 g trigger force, 5 s interval between the compressions, and 200 pp/s data acquisition rate. TA-DGA model fixture was used for tensile properties testing. The samples were measured with a starting distance of 60 mm and target distance of 50 mm. The trigger force was set at 10 g with a tensile speed of 2 mm/s. Measurements were performed in six replicates.

### Statistical analysis

The results are reported as mean and standard deviation of at least triplicate. The statistically analysis was performed by variance analysis (ANOVA) using SPSS 17.0 statistical software (SPSS Inc., Chicago, IL, USA). Significant differences between the means were determined by Duncan test (*P* < 0.05). Pearson's correlation coefficients among parameters were also calculated using SPSS 17.0 statistical software.

## Results and discussion

### Properties of rice flour

#### Compositions of rice flour

Total starch, amylose, crude protein and fat contents of different rice flours are shown in Table 1. the basic physical and chemical indexes of different rice varieties are different. Starch and protein are main rice compositions. The total starch and protein content in rice flour were 73.08–75.09 g/100 g (GC>SM>ZG>SG) and 7.31–8.04 g /100 g (GC>SM>SG>ZG), respectively. The amylose contents were in the range of 21.00–23.91 g/100 g following the order of GC>ZG>SG>SM. There were significant differences in amylose and protein content among different rice flours (*P* < 0.05).

**Table 1:** Physicochemical compositions of different rice flour.

**Rice varieties**	**Moisture (%)**	**Total starch (%)**	**Amylose (%)**	**Protein (g/100 g)**	**Fat (g/100 g)**
ZG	10.82 ± 0.57^ab^	74.31 ± 0.07^b^	22.44 ± 0.15^c^	7.31 ± 0.00^a^	0.82 ± 0.02^a^
SG	10.11 ± 0.42^a^	73.08 ± 0.08^a^	21.96 ± 0.08^b^	7.45 ± 0.00^b^	1.03 ± 0.02^b^
GC	10.98 ± 0.42^bc^	75.09 ± 0.32^c^	23.91 ± 0.12^d^	8.04 ± 0.00^d^	1.01 ± 0.01^b^
SM	11.73 ± 0.16^c^	74.46 ± 0.31^a^	21.00 ± 0.10^a^	7.74 ± 0.01^c^	1.50 ± 0.01^c^

ZG, zhengui; SG, shuanggui; GC, guichao; SM, suimi.

Means followed by different letters in the same column are significantly different at P < 0.05.

#### Solubility and swelling power of rice flour

The solubility (S) and swelling power (SP) are shown in Table 2. The lower S is associated with smaller cooking loss of rice noodles, and low SP of starch granules relates to relatively high anti-shear ability (27). Amylose content in ZG, SG and GC rice flours has no significant correlation with S, which was in agreement with previous reports. Li and Vasanthan suggested that samples with higher amylose content were less susceptible to swelling during gelatinization (28). Jiao et al. reported that starch-based material of pea starch forms a stronger gel due to a higher amylose content of pea starch, which is desirable in noodle processing (29). However, compare with ZG, SG, and GC, the solubility of SM rice flour increased significantly (*P* < 0.05) and the swelling power of SM rice flour decreased significantly (*P* < 0.05) with the amount of amylose decreased to 21.00%. The reason may be that S and SP were not only related to amylose content, but also related to the structure of amylopectin. Previous studies suggested that the value of SP depends on the magnitude of interaction between starch chains within the crystalline and amorphous domains (30). The SP of starch mainly reflects the insolubility of amylopectin, which is primarily caused by the formation of hydrogen bond between side chains of amylopectin (31). The expansion characteristic of amylopectin is also related to the length of the amylopectin chains (32). When rice amylose contents are close, contents of protein and fat, and damaged starch content that was caused during milling process may take a primary role in influencing the characteristics of rice gel (10–12). Tong et al. (2) found that physicochemical characteristics (the degree of starch damage, etc.) of rice flours prepared from wet-, dry- and semidry-milling methods were different. According, the resulting textural profile and cooking qualities of rice noodles prepared with these different milled rice flours were varied significantly.

**Table 2:** Physicochemical properties of different rice flour.

**Variety**	**Adhesive strength (mm)**	**Solubility (%)**	**Swelling power (g/g)**	**Thermal properties**						**Pasting properties**				
				**To (°C)**	**Tp (°C)**	**Tc(°C)**	**ΔH_1_ (J/g)**	**ΔH_2_ (J/g)**	**ΔH/%**	**PV (cP)**	**TV (cP)**	**FV (cP)**	**BD (cP)**	**SB (cP)**
ZG	42.83 ± 0.76	4.53 ± 0.46^a^	10.06 ± 0.99^b^	64.43 ± 0.12^a^	69.32 ± 0.17^a^	78.39 ± 0.22^a^	9.75 ± 0.10^a^	3.08 ± 0.00^a^	31.61 ± 0.31^a^	250.00 ± 3.00^c^	246.33 ± 2.08^c^	401.33 ± 1.53^b^	3.67 ± 1.15^a^	155.00 ± 1.73^a^
SG	46.10 ± 0.36	4.58 ± 0.87^a^	10.47 ± 0.42^b^	72.90 ± 0.36^b^	77.76 ± 0.18^c^	84.41 ± 0.23^c^	11.86 ± 0.26^c^	5.55 ± 0.10^b^	46.78 ± 0.54^b^	195.33 ± 1.53^b^	193.33 ± 1.53^b^	376.00 ± 4.36^a^	2.00 ± 0.00^a^	182.67 ± 3.06^b^
GC	35.00 ± 0.20	4.48 ± 0.25^a^	9.85 ± 0.37^b^	73.07 ± 0.07^b^	77.08 ± 0.08^b^	83.68 ± 0.08^b^	11.42 ± 0.02^b^	5.96 ± 0.06^c^	52.19 ± 0.43^d^	365.25 ± 2.63^d^	363.00 ± 2.94^d^	604.00 ± 5.77^d^	2.25 ± 0.50^a^	241.00 ± 6.78^d^
SM	49.50 ± 0.78	5.72 ± 0.12^b^	8.66 ± 0.24^a^	75.36 ± 0.27^c^	79.86 ± 0.18^d^	94.26 ± 0.18^d^	13.40 ± 0.26^d^	6.51 ± 0.09^d^	48.62 ± 0.99^c^	184.25 ± 0.50^a^	181.50 ± 1.91^a^	411.50 ± 6.45^c^	2.75 ± 1.71^a^	230.00 ± 5.94^c^

ZG, zhengui; SG, shuanggui; GC, guichao; SM, suimi.

To, onset temperature; Tp, peak temperature; Tc, final temperature; ΔH_1_, gelatinization enthalpy; ΔH_2_, regenerative enthalpy; ΔH, The retrogradation degree.

PV, peak viscosity; TV, trough viscosity; FV, final viscosity; BD, breakdown; SB, setback.

Means followed by different letters in the same column are significantly different at P < 0.05.

#### Thermal properties of rice flour

The results of thermal properties are shown in Table 2. There were significant differences of thermal properties (T_P_, T_C_, and ΔH_1_) in the four rice raw materials (*P* < 0.05). T_P_, T_C_ and ΔH_1_ of rice followed the order of SM>SG>GC>ZG. There were also significant differences of ΔH_2_ between the four rice raw materials following the order of SM>GC>SG>ZG (*P* < 0.05). According to literature, gelatinization temperature is associated with internal arrangement of starch granules, and crystallinity degree affects gelatinization enthalpy (30). The structural changes in starch granules, including the interactions of amylose-lipid and amylose-amylose, could lead to changes in gelatinization properties (33). Therefore, a slight decline in T_p_ might be attributed to the arrangement of starch granules, the difference in crystallinity degree, and the interactions of amylose-amylose, amylose-lipid and amylose-protein in granules. The variation in chain-length distribution in amylopectin might also account for the evident increase of ΔH_1_, because more energy was needed to dissociate longer linear chains (34, 35). These changes in gelatinization properties directly affected the cooking quality, as lower Tp meant shorter cooking time (36).

The results of retrogradation properties are also shown in Table 2. It is known that, retrogradation is an inevitable procedure during rice noodle processing (37). In this study, the retrogradation degree (ΔH) of different rice materials were significantly different among different varieties (31.61–52.19%). The ΔH values from high to low followed the order of GC (52.19%) >SM (48.62%) >SG (46.78%) >ZG (31.61%). Research showed that ΔH is highly correlated with amylose content and starch sources (35). In this study, GC had a higher ΔH than other three rice flours (Table 2), which may due to its higher amylose content (Table 1). However, it is interesting that when the amylose contents were close (ZG, SG, and SM), there was no correlation between the ΔH and the amylose. In this study, the amylose content in ZG rice was slightly higher than that in SG and SM, but its ΔH was the lowest, which may be affected by other factors such as protein or fat. The content of protein and fat in ZG rice is relatively low compared to other varieties. Our results were in agreement with Marcoa and Likitwattanasade et al. (38, 39), who found that ΔH increased with protein addition. Thus, when choosing raw materials for rice flour processing, the influence of amylose content should be considered first for the acute improvement of retrogradation degree. Meanwhile the influence of other ingredients such as proteins and fats content should be taken into account when amylose content were similar.

#### Pasting properties of rice flour

**Figure 1:**
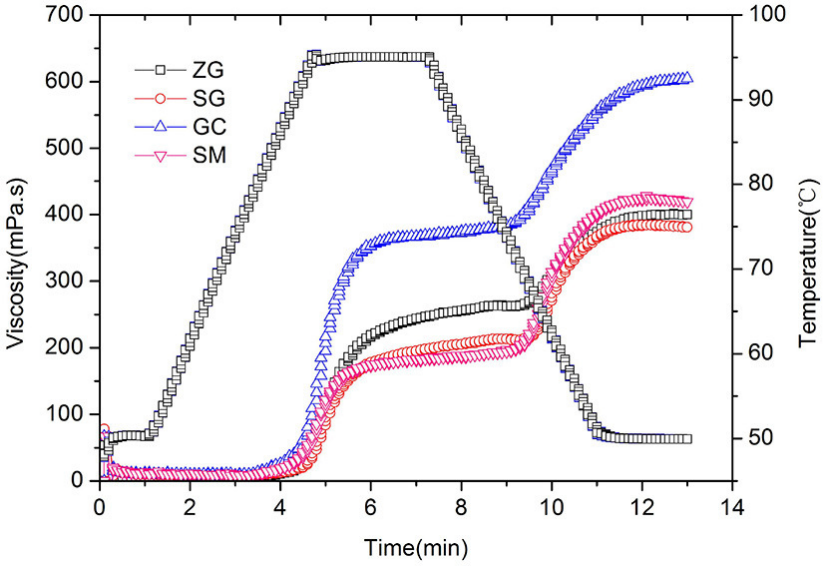
Pasting properties of rice flour. ZG, zhengui; SG, shuanggui; GC, guichao; SM, suimi.

Pasting properties of starches can be used to estimate the applicability of rice noodle making (40). The pasting properties of different rice starch are presented in Table 2 and Figure 1. All samples exhibited a typical pasting property of native rice flour, which contained peak viscosity during heating and subsequent breakdown on holding at 95°C, followed by setback during cooling. The peak and trough viscosities of rice flour samples followed the same order of GC>ZG>SG>SM. Peak viscosity (PV) is the maximum viscosity obtained from gelatinized starch during heating in water, which indicates the water-binding capacity of the starch granules (19). High PV of rice batter enhances its adhesion properties (41). Through viscosity (TV) refers to the viscosity decreasing rapidly after reaching the peak and falling to the lowest viscosity at a high temperature. PV and TV had a positive correlation with amylose content (*P* < 0.05). Final viscosity (FV) is the viscosity of sample at the end of the test at 50°C. Pearson's correlation analysis showed that the FV of rice flours was not positive correlated with amylose content (*P* > 0.05). FV of GC was noticeably higher than SM and ZG followed by SG. the FV is not positive correlation with amylose content, as can be seen from Table 2. FV value of SM was larger than ZG and SG, the reason may be related to varieties, SM used in this study was the broken rice of GC varieties. The result of FV was contradictory to the previous study (20), Wu et al. reported that high FV was accompanied by high gel hardness. The breakdown (BD) indicates the propensity of starch granules for disintegration (42) and represents hot paste stability. BD was caused by structural disruption of gelatinized starches at high temperature and affected by amylose content and fine structure of amylopectin (19, 43). Among the four cultivars, BD had no significant difference and was not directly correlated with the amylose content. The setback (SB) determines the retrogradation tendency of the product and reflects short-term aging ability and cold paste stability of starch (19, 44). SB is affected by content and molecular size of amylopectin in a pure starch system (34). In a complex flour system, SB could be influenced by starch content, amylose: amylopectin ratio, structural characteristics of amylose, and other factors like protein and lipid contents.

#### Rheological properties of rice flour

**Figure 2:**
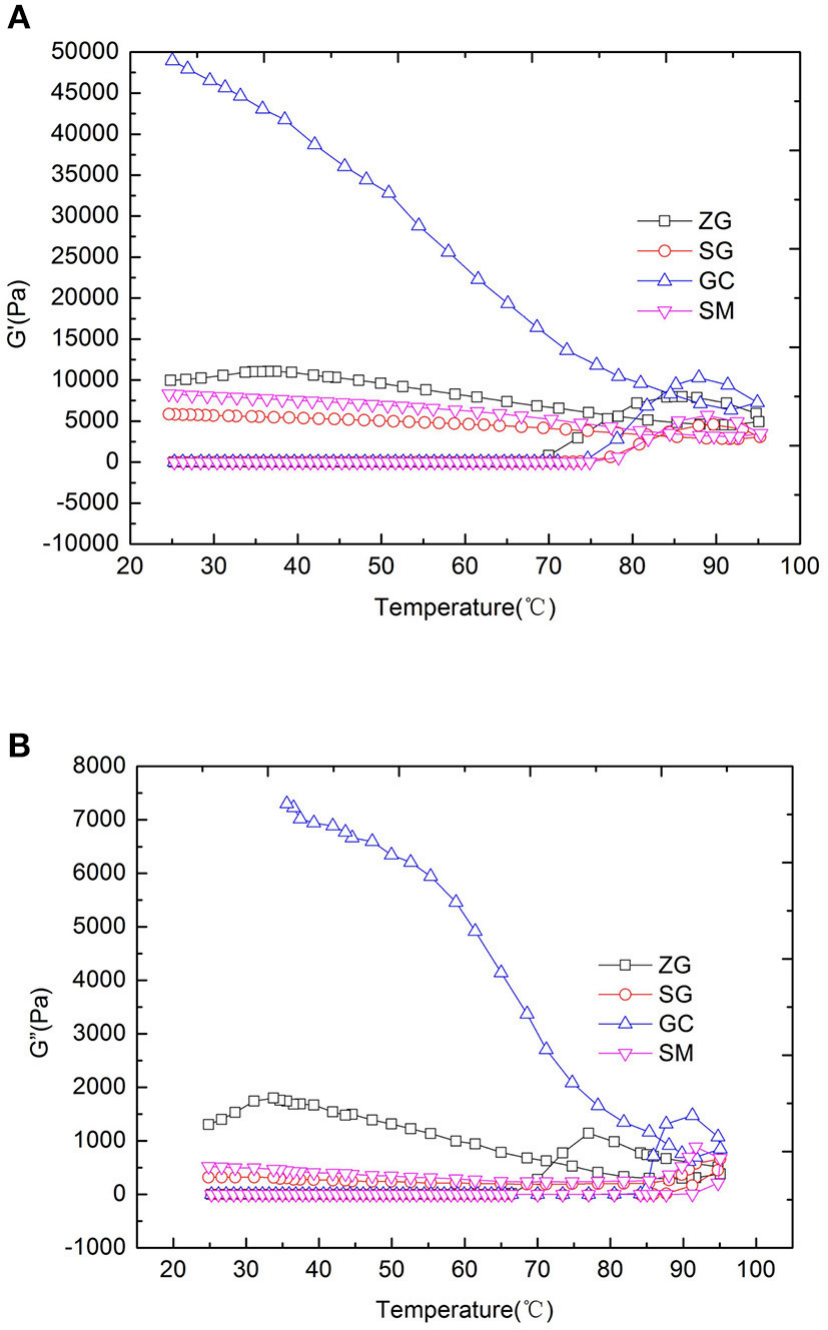
Rheological properties of different rice flours samples in the process of temperature ramp **(A)** for the G′ of samples, **(B)** for the G″ of samples. G′, storage modulus; G″, loss modulus; ZG, zhengui; SG, shuanggui; GC, guichao; SM, suimi.

**Figure 3:**
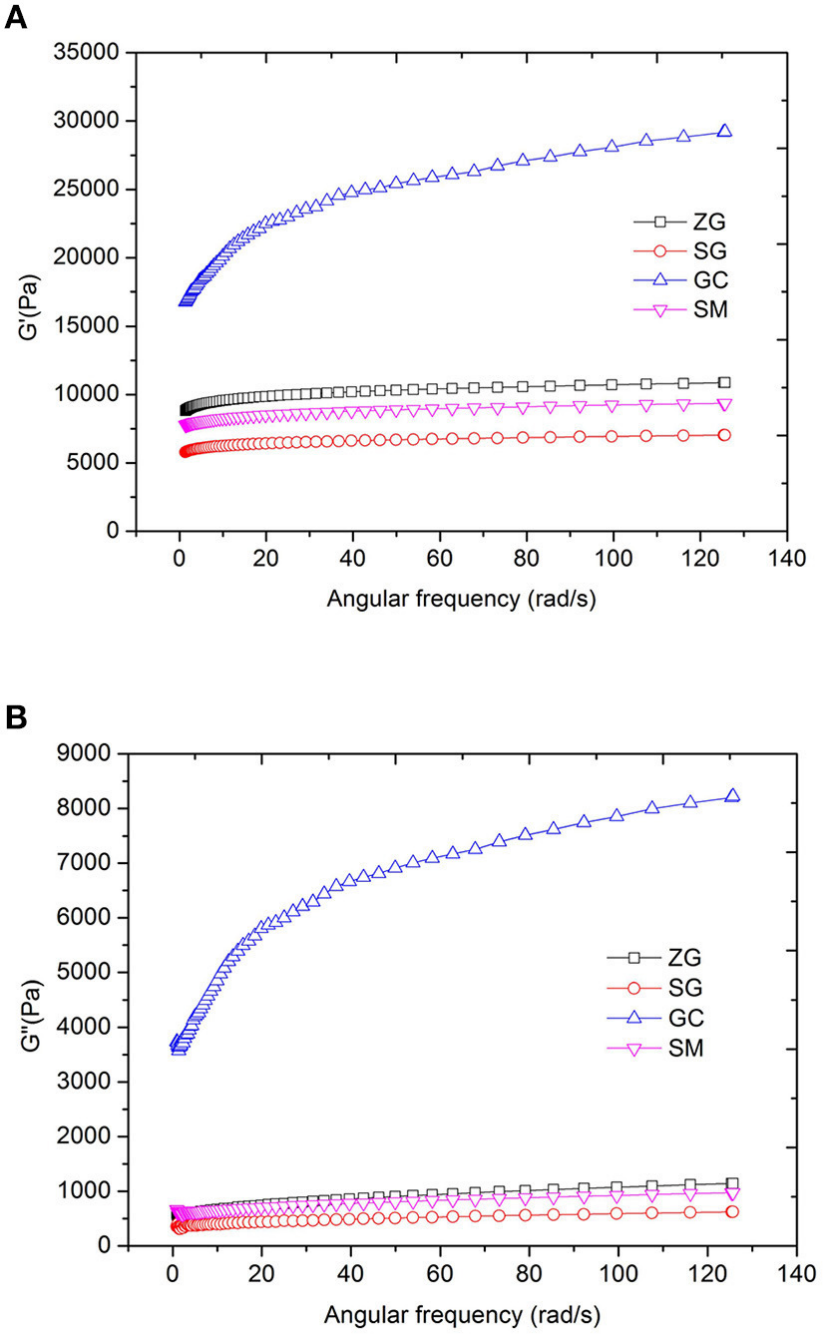
Rheological properties of different rice flours samples in the process of frequency sweep **(A)** for the G′ of samples, **(B)** for the G“ of samples). G′, storage modulus, G″, loss modulus, ZG, zhengui; SG, shuanggui; GC, guichao; SM, suimi.

Rice flours that were prepared from different rice cultivars had different rheological properties during a heat-cooling cycle in a temperature range of 25–95°C (Figure 2A). During heating, G′ and G″ sharply increased at ~70°C. When the temperature reached 80–85°C, G′ and G″ reached the maximum values, which was caused by swelling and gelatinization of starch granules (45). With the continuous increases of temperature, G′ and G″ began to decrease rapidly reaching the highest temperature, which was due to the deformation of swollen starch granules and breakdown of crystalline structure after treatment (36, 45). During cooling from 95 to 25°C, G′ and G″ increased steadily. During this process, starch granules aggregated, and association formed between starch and other molecules (30, 46). G′ and G″ values were higher for the rice flour samples with higher amylose contents. As can be seen in Figures 2A, 3A, the G′ and G″ values of four kinds of rice were as follows: GC>ZG>SG>SM, which was in accordance with the results obtained by Charles et al. (47) who found that high contents of amylose contribute to gel firmness and stability. In addition, it is interesting to note the changes of G′ were not linear with the increases of amylose. Differences of amylose content between SG (21.96%) and SM (21.00%) resulted in small differences in G′, but the increases of amylose content to 23.91% (GC) seems to result in a large G′ value. The higher the amylose content of rice flour, the desirable the viscoelasticity of the gel system.

The gelatinized rice flour formed a strong gel (Figure 3). G′ had a notably higher value than G“ and had a weak dependence on frequency, suggesting a dominant elastic rheological behavior. G′ and G″ increased with increasing amylose content. Amylose has fewer branches than amylopectin, and it can retrogradation in a shorter time to form molecular aggregations and intermolecular double helices. Starch with a higher amylose content tends to form a stronger gel in a shorter time (47). Gels with higher G′ showed higher rigidity and strength (45, 48). Amylose content and starch type affect the viscoelasticity of gels (49). Extruded rice noodle is a kind of gel product, so the rheological analysis can be used as an auxiliary approach for selecting raw rice materials for noodle production.

### Rice noodles qualities

#### Cooking qualities and sensory evaluation of rice noodles

**Figure 4:**
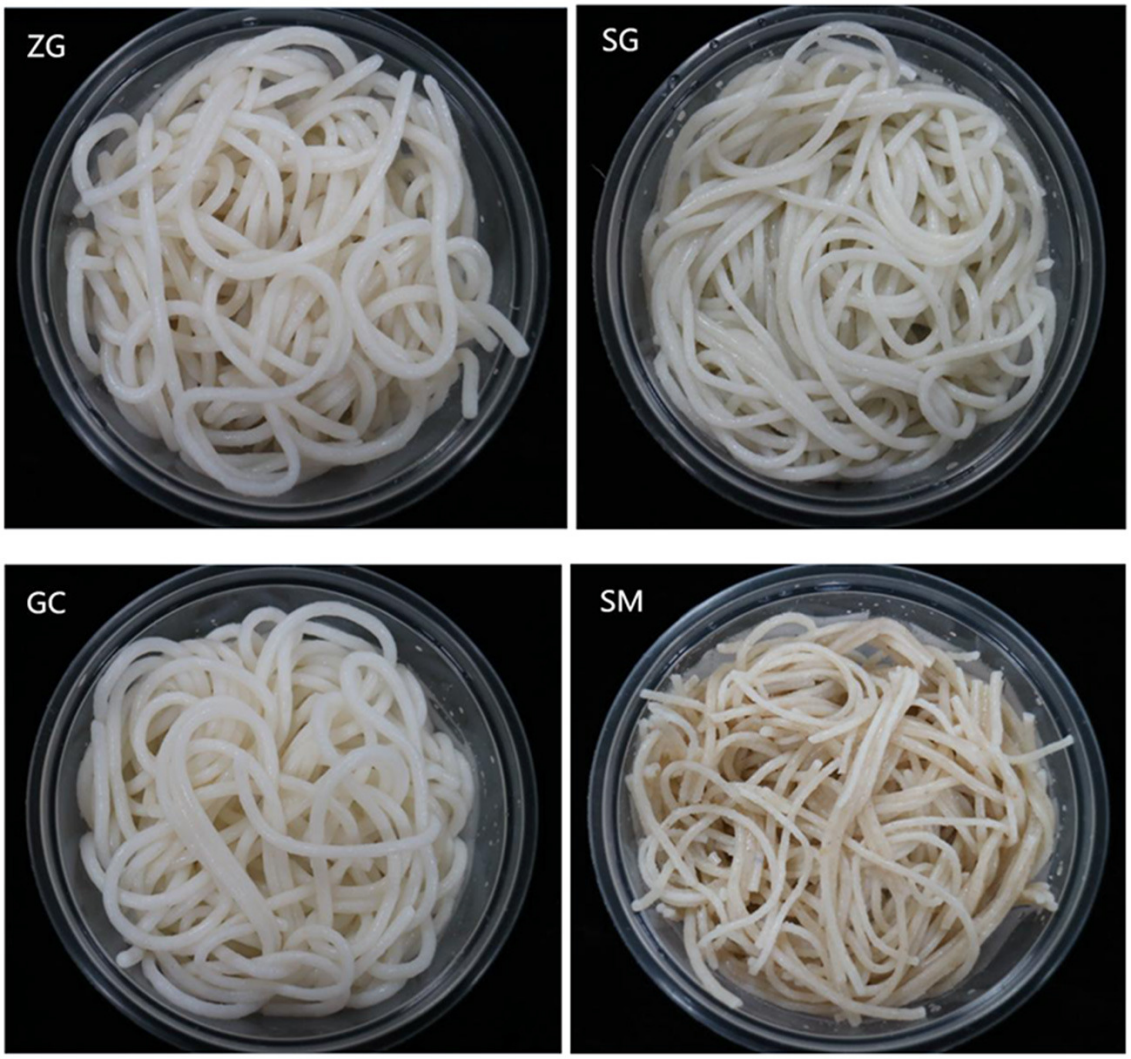
Morphology of rice noodles made from selected varieties of early polished indica rice. ZG, zhengui; SG, shuanggui; GC, guichao; SM, suimi.

Rehydration time, broken rate, and cooking loss of rice noodles are shown in Table 3. The rehydration time is associated with its cooking time, and the broken rate and cooking loss were two important indexes of the cooking qualities for rice noodles. ZG, SG, and GC had no significant difference in rehydration time (15–16 min, *P* > 0.05), which was significantly higher than SM (12 min). SM had approximately two times of broken rate higher than ZG, SG, and GC, while the latter three had no significant differences (*P* > 0.05). The cooking loss of rice noodles followed the order of GC<ZG<SG<SM. Overall, GC and ZG noodles had the best cooking quality with low broken rate and cooking loss, followed by SG. SM had the lowest cooking quality with highest broken rate and cooking loss. Previous research had reported that noodles with a higher amylose content generally had a shorter rehydration rate, higher gel strength, and smaller breaking rate and cooking loss (35, 37). In this paper, the similar phenomenon were observed. The morphology of rice noodles shown in Figure 4 was in agreement with the rice noodle quality analysis (Table 3). There were only slight differences of the rice noodles morphology among ZG, SG, and GC, as their starch granular structure was not destroyed during processing. The appearance of ZG, SG, and GC had negligible differences, but SM noodle had a darker color and more short segments, and the shape of rice noodles produced by SM was relatively poor. The reason may be the overall structure of the SM rice starch granules was damaged worse during the process of shucking and stripping.

**Table 3:** Quality results of different rice noodles.

**Variety**	**Rehydration time (min)**	**Cooking quality**		**TPA**			**Tensile properties**		**Sensory score**						
		**Broken rate (%)**	**Cooking loss (%)**	**Hardness (g)**	**Springiness (mm)**	**Chewiness (mJ)**	**Peak load (g)**	**Deformation at peak (mm)**	**Color**	**Odor**	**Tissue shape**	**Firmness**	**Smoothness**	**Elasticity**	**Total score**
ZG	15.33 ± 0.58^b^	10.33 ± 1.53^a^	14.57 ± 0.31^a^	1084.00 ± 175.74^a^	1.53 ± 0.09^c^	14.72 ± 2.49^b^	30.20 ± 11.82^a^	13.58 ± 4.32^ab^	13.00 ± 1.58^b^	9.00 ± 0.71^a^	13.00 ± 1.41^b^	16.00 ± 0.71^c^	18.00 ± 1.00^b^	17.00 ± 1.58^c^	86.00 ± 2.45^c^
SG	15.00 ± 1.00^b^	10.33 ± 4.73^a^	16.33 ± 0.42^b^	1097.00 ± 190.50^a^	1.36 ± 0.03^b^	12.37 ± 2.41^b^	26.00 ± 5.29^a^	11.28 ± 4.09^ab^	12.00 ± 1.58^b^	9.00 ± 0.71^a^	13.00 ± 1.58^b^	14.00 ± 1.58^b^	16.00 ± 1.58^b^	14.00 ± 1.58^b^	78.00 ± 3.32^b^
GC	15.67 ± 0.58^b^	9.00 ± 1.00^a^	14.33 ± 0.42^a^	1628.67 ± 64.04^b^	1.55 ± 0.03^c^	21.53 ± 3.21^c^	56.00 ± 14.51^b^	17.16 ± 4.38^b^	11.00 ± 1.41^b^	8.00 ± 0.71^a^	13.00 ± 1.00^b^	18.00 ± 0.71^d^	17.00 ± 1.00^b^	17.00 ± 0.71^c^	84.00 ± 2.74^c^
SM	12.00 ± 1.00^a^	22.00 ± 2.65^b^	25.50 ± 0.50^c^	941.67 ± 214.72^a^	0.95 ± 0.06^a^	5.70 ± 1.32^a^	19.60 ± 5.55^a^	9.95 ± 4.13^a^	8.00 ± 1.22^a^	8.00 ± 0.71^a^	10.00 ± 2.24^a^	11.00 ± 0.71^a^	13.00 ± 2.24^a^	12.00 ± 1.58^a^	62.00 ± 4.30^a^

Means followed by different letters in a column are significantly different at P < 0.05.

The results of sensory evaluation are summarized in Table 3. There were no significant difference (*P* > 0.05) in odor of ZG, SG GC, and SM. While compared with noodles made from SM, there were significant increases in the color, tissue shape and smoothness points of noodles made by GC, ZG, and SG (*P* < 0.05). The firmness points were correlated with rice amylose content. As we can see in Table 3, firmness points significantly increased (*P* < 0.05) when amylose content of different rice increase from 21.00 to 23.91 % (GC>ZG>SG>SM). Elasticity points were also correlated with rice amylose content. Compared with noodles made from SM, there were significant increases elasticity points of noodles made by GC, ZG, and SG (*P* < 0.05). However, elasticity points of ZG and GC had no significantly differences (*P* > 0.05). The total score was calculated from the sum of color, odor, tissue shape, firmness, smoothness and elasticity scores. Total scores from high to low were: ZG, GC, SG, and SM. The total scores were not completely positive correlated with rice amylose content. As we can see, though the amylose content of GC rice (23.91%) was slightly higher than that of ZG rice (22.44%), its score (84.00) was not higher than that of ZG rice (86.00) (*P* > 0.05). Therefore, when the amylose content of different rice varieties were approximately, in addition to considering the main factor of starch, the influence of other factors such as protein also should be properly considered.

#### Texture properties of rice starch noodles

The texture of cooked noodles has substantial effects on the sensory properties and the resulting consumers' acceptance. In this study, noodle texture was analyzed using a compression test (texture profile analysis) and a tensile test (Table 3). In TPA, hardness is a measurement of force to compress the sample in the first bite with molar teeth. Springiness is the degree to which a sample returns to its original shape after partial compression with the molar teeth, and chewiness is the amount of work to chew the sample to get it ready to swallow. Chewiness is related to hardness, cohesion and elasticity, and it equals to product of hardness, cohesiveness and springiness. When the amylose content of GC rice was 23.91%, the hardness of the rice starch noodles was 1628.67 g and significantly higher (*P* < 0.05) than that of noodles made from ZG, SG, SM (with hardness of 1084.00, 1097.00, and 941.67 g, respectively), which is likely related to amylose content. The amylose content (23.91%) in GC rice flour had a higher amylose content than ZG, SG and SM, and GC starch formed a stronger gel strength than the other three (Table 1, Figures 2, 3). The hardness of ZG, SG and SM noodles had no significant differences (*P* > 0.05). In addition, the changes of springiness, chewiness, peak load and deformation at peak value of rice noodles were consistent with the changes of amylose content. There was no significant difference between GC and ZG in springiness, which was consistent with G′ (Table 3, Figures 2A, 3A). There was no significant difference of chewiness value between ZG and SG (*P* >0 .05). For the deformation at peak, there was no significant difference in the variation of other rice varieties in addition to SM, which indicated that although the amylose content of rice was dominant, the texture quality of rice flour also might be affected by other factors especially when the amylose content of different rice varieties are similar.

In summary, the texture profile of different rice noodles varied mainly depending on amylose content. Early indica rice of GC with higher amylose content (23.91%) was more suitable to produce rice noodles with higher elasticity, such as snail noodles. ZG and SG indica rice with moderate amylose content was more suitable for processing relatively soft taste of rice noodles such as rolled rice noodles and sliced rice noodles. In addition, although the textural properties of rice noodle made by SM was relatively poor, it can be mixed with some high amylose rice in food industry, which reduces production cost and increases quality of rice noodles.

#### Correlations between physicochemical properties of rice starch and qualities of rice noodles

Correlations between physicochemical properties of rice flour and the qualities of rice noodles were analyzed. Pearson's correlation coefficients are presented in Table 4. The physicochemical properties of rice flour had a significant influence on the qualities of rice noodles. Amylose content of the rice had a positive correlation with hardness of rice noodles (*P* < 0.5) and a strong positive correlation with peak and trough viscosities, chewiness, peak load and deformation at peak of rice starch noodles (*P* < 0.01). Adhesive strength had a strong negative correlation with hardness, chewiness, peak load and deformation at peak of rice starch noodles (*P* < 0.01). In addition, there were positive correlations between RVA paste viscosities and texture qualities of rice starch noodles (*P* < 0.01). PV and TV had positive correlations with chewiness (*P* < 0.05), and a strong positive correlation with hardness, peak load and deformation at peak of rice starch noodles (*P* < 0.01). FV also showed a positive correlation with hardness and peak load (*P* < 0.05). This was consistent with what was reported by Bhattacharya et al. (50). Who suggested the suitability and advantages of using pasting properties for selection of rice cultivars suitable for noodle preparation. The rice noodle properties in this study, including the cooking qualities, texture properties, and sensory evaluation score, were closely correlated with amylose content, adhesive strength, and pasting properties. Meanwhile, other influencing factors (e.g., protein) should also be considered.

**Table 4:** Correlations between physicochemical properties of rice starch and rice starch noodles qualities.

	**Amylose**	**Protein**	**Adhesive strength**	**Solubility**	**Swelling power**	**ΔH**	**PV**	**TV**	**FV**	**BD**	**SB**	**Broken rate**	**Cooking lost**	**Hardness**	**Springiness**	**Chewiness**	**Peak load**	**Deformation at peak**	**Sensory score**
Amylose	1																		
Protein	0.45	1																	
Adhesive strength	−0.99^**^	−0.51	1																
Solubility	−0.77	0.16	0.71	1															
Swelling power	0.49	−0.41	−0.4	−0.92^**^	1														
ΔH	0.13	0.83	−0.15	0.26	−0.29	1													
PV	0.96^**^	0.62	−0.98^**^	−0.57	0.23	0.22	1												
TV	0.96^**^	0.62	−0.98^**^	−0.57	0.23	0.23	1.00^**^	1											
FV	0.83	0.86	−0.87	−0.29	−0.04	0.52	0.93^**^	0.93^**^	1										
BD	−0.15	−0.51	0.12	0.06	−0.18	−0.86	−0.09	−0.1	−0.29	1									
SB	0.18	0.96^**^	−0.24	0.42	−0.59	0.89^**^	0.37	0.37	0.68	−0.54	1								
Broken rate	−0.79	0.12	0.73	1^**^	−0.91^**^	0.21	−0.60	−0.60	−0.33	0.11	0.37	1							
Cooking loss	−0.81	0.14	0.75	0.99^**^	−0.88	0.30	−0.63	−0.63	−0.34	−0.01	0.41	0.99^**^	1						
Hardness	0.95^**^	0.69	−0.96^**^	−0.59	0.30	0.43	0.96^**^	0.96^**^	0.94^**^	−0.37	0.46	−0.63	−0.62	1					
Springiness	0.85	−0.08	−0.81	−0.97^**^	0.80	−0.32	0.71	0.70	0.42	0.09	−0.40	−0.97^**^	−0.99^**^	0.66	1				
Chewiness	0.99^**^	0.35	−0.98^**^	−0.84	0.58	0.06	0.92^**^	0.92^**^	0.76	−0.14	0.07	−0.86	−0.87	0.92^**^	0.90^**^	1			
Peak load	0.97^**^	0.65	−0.98^**^	−0.61	0.30	0.33	0.99^**^	0.99^**^	0.94^**^	−0.25	0.41	−0.64	−0.65	0.99^**^	0.70	0.94^**^	1		
Deformation at peak	0.99^**^	0.49	−1.00^**^	−0.69	0.36	0.09	0.99^**^	0.99^**^	0.86	−0.04	0.22	−0.71	−0.74	0.94^**^	0.81	0.97^**^	0.97^**^	1	
Score	0.80	−0.17	−0.75	−0.96^**^	0.81	−0.41	0.64	0.64	0.33	0.17	−0.50	−0.95^**^	−0.99^**^	0.58	0.99^**^	0.86	0.63	0.76	1

^*^, ^**^significant at P <0.05, P <0.01, respectively.

## Conclusions

The relationships between the characteristics of four rice raw materials, processing performance, and cooking and sensory properties of rice noodles were discussed in the present study. This study shows that amylose content, adhesive strength, and pasting properties had great influences on cooking qualities and sensory properties of rice noodles. Starch properties could be used for selection of suitable rice materials for noodle production and prediction of rice noodle quality. This study is useful for selecting rice for food industry in different regional markets for specific rice noodle product requirements. It provides information for formulating pre-extrusion material for rice noodle production by combining several dried rice flours, such as early indica rice, late indica rice and some plant starches with higher amylose than starch (e.g., corn starch). Further study could focus on optimizing rice noodle processing conditions and producing rice noodles with regional characteristics and high quality.

## Data availability statement

The original contributions presented in the study are included in the article/supplementary material, further inquiries can be directed to the corresponding author/s.

## Author contributions

PW: methodology, investigation, data curation, and writing—original draft. FF: investigation and writing—review and editing. GL: writing—review and editing. YZ: conceptualization, validation, and writing—review and editing. LW and KZ: software. XY: investigation, conceptualization, and supervision. YW: validation. ML: formal analysis. JS and SD: contributed helpful discussion and scientific advice during the preparation of manuscript. All authors contributed to the article and approved the submitted version.

## Funding

This research was supported by Science and Technology Major Project of Guangxi (Grant Nos. Gui Ke AB21220045, AB21196067, and AA17202029), the Special Fund for Agro-Scientific Research in the Public Interest (Grant No. 201503001-6), Guangxi Science and Technology Pioneer Project (Grant No. 202115), Special Fund for Guangxi Bagui Scholars (Grant No. [2016]21), Foundation of Fundamental Research Project from Guangxi Academy of Agricultural Sciences (Grant Nos. JZ202019 and 2021JM103) and Dominant Discipline Team Project (Grant Nos. Gui Nong Ke 2015YT87 and 2018YM05).

## Conflict of interest

The authors declare that the research was conducted in the absence of any commercial or financial relationships that could be construed as a potential conflict of interest.

## Publisher's note

All claims expressed in this article are solely those of the authors and do not necessarily represent those of their affiliated organizations, or those of the publisher, the editors and the reviewers. Any product that may be evaluated in this article, or claim that may be made by its manufacturer, is not guaranteed or endorsed by the publisher.
